# D2D-Assisted Multi-User Cooperative Partial Offloading in MEC Based on Deep Reinforcement Learning

**DOI:** 10.3390/s22187004

**Published:** 2022-09-15

**Authors:** Xin Guan, Tiejun Lv, Zhipeng Lin, Pingmu Huang, Jie Zeng

**Affiliations:** 1 School of Information and Communication Engineering, Beijing University of Posts and Telecommunications (BUPT), Beijing 100876, China; 2 Key Laboratory of Dynamic Cognitive System of Electromagnetic Spectrum Space, College of Electronic and Information Engineering, Nanjing University of Aeronautics and Astronautics (NUAA), Nanjing 211106, China; 3 School of Artificial Intelligence, Beijing University of Posts and Telecommunications (BUPT), Beijing 100876, China; 4 School of Cyberspace Science and Technology, Beijing Institute of Technology, Beijing 100081, China

**Keywords:** mobile edge computing, D2D communication, partial offloading, Q learning, deep Q-network

## Abstract

Mobile edge computing (MEC) and device-to-device (D2D) communication can alleviate the resource constraints of mobile devices and reduce communication latency. In this paper, we construct a D2D-MEC framework and study the multi-user cooperative partial offloading and computing resource allocation. We maximize the number of devices under the maximum delay constraints of the application and the limited computing resources. In the considered system, each user can offload its tasks to an edge server and a nearby D2D device. We first formulate the optimization problem as an NP-hard problem and then decouple it into two subproblems. The convex optimization method is used to solve the first subproblem, and the second subproblem is defined as a Markov decision process (MDP). A deep reinforcement learning algorithm based on a deep Q network (DQN) is developed to maximize the amount of tasks that the system can compute. Extensive simulation results demonstrate the effectiveness and superiority of the proposed scheme.

## 1. Introduction

In recent years, with the development of wireless networks and the popularity of smart mobile devices, mobile applications such as augmented reality (AR), virtual reality (VR), and facial recognition payment have grown exponentially [1,2]. These applications tend to be computation intensive and require low latency, but the battery capacities, computation resources, and storage capacities of mobile user equipment (UE) are very limited. As a result, most emerging applications may not be suitable for local execution on mobile devices [3,4]. To address this daunting challenge, the functions of the central network are increasingly moving towards the edge of the network [4].

Mobile edge computing (MEC) is regarded as a promising paradigm. This technique moves service platforms with computing, storage, and communication capabilities to the edge node (base station (BS)) nearest to the mobile devices [5,6,7,8,9]. MEC allows resource-constrained mobile terminals to migrate part or all of the complex applications to the edge cloud, becoming a low-latency, low-energy, and efficient solution [10,11,12]. However, the heterogeneous characteristics of BSs and MEC servers and the limited resources of communication and edge computing bring challenges to computational offloading methods [13].

Computing offloading is one of the key issues in MEC [14,15]. Its main task is to plan the offloading scheme of computing tasks and the allocation scheme of computing resources to reduce delays, save energy consumption, and improve computing resource utilization. There are two main task offloading strategies. In full offloading, each task can be completely offloaded to the resource device or run completely locally [2,16,17]. In partial offloading, each task can be partially offloaded to the resource device and partially left local [18,19,20].

Although the above studies have demonstrated the advantages of MEC computation offloading for improving wireless network computing performance, the limited computing resources of BSs are not always sufficient to support all mobile devices within their coverage. To solve this problem, some work studies offload computation to the neighboring devices through device-to-device (D2D) communication links [21,22,23,24,25]. The authors of [21] believe that each mobile user has its corresponding mobile peer; thus, the computing task can be partially offloaded to the edge cloud or its mobile peer. Successive convex approximation (SCA) and geometric programming (GP) are used to solve the energy minimization problem. Reference [22] solves the overall energy consumption problem of the system under the three-layer network architecture integrating cloud computing, MEC, and D2D communication through game theory. Reference [23] regards one near computing device with sufficient resources near each mobile user as a distributed computing node (DCN), and the mobile user can connect to the associated DCN through D2D. The problem of complete offloading of computing tasks between local, edge cloud, and DCN is studied relying on game theory, thereby reducing the delay and energy consumption of the system.

Although the above-mentioned research results have laid a solid foundation for utilizing the computing resources of idle devices in the MEC, the utilization efficiency of resources on idle devices has yet to be improved. By assuming that each mobile user is associated with a distributed computing node (DCN), Ref. [23] does not consider the DCN selection. Ref. [20] uses idle devices for D2D-assisted transmission and optimizes transmission scheduling, which does not use idle device resources for computing assistance. Although [25,26,27] optimizes the pairing between TD and RD, they stipulate that each device with an idle resource can only offer a D2D offloading service to one other device at most, and limits the pairing method between devices to one to one. Ref. [28] utilizes the resources of idle devices for computing assistance through D2D communication, and assumes that each idle device can accept multiple computing tasks. However, complete offloading is considered in this paper. Additionally, Ref. [28] assumes that when multiple computation tasks are offloaded to the same idle device, the tasks will share the computation resource equally.

Most of the works on MEC computing offload are aimed at reducing latency, saving energy, reducing energy efficiency, etc., and many state-of-the-art works only consider integrating multiple indicators for optimization [27,29]. However, the computing capability of the system can also be determined by the number of devices the system can serve. Because reduction in latency is often accompanied by the consumption of computing resources in the system, the number of devices supported by the system can be maximized subject to the constraint of maximum delay. In this way, the utilization efficiency of computing resources can be further improved without affecting user experience. As a result, the proposed scheme can cope with the continuous increase in the number of devices in the 6G network. In addition, traditional methods, such as dynamic programming, game theory, and GP, are often used to reduce the delay and energy consumption in full offloading, since such problems can be expressed as mixed-integer programming (MIP).

Compared with traditional optimization methods, reinforcement learning (RL) can deal with more complex MEC problems [30]. Reference [16] uses Q-learning and a deep Q network (DQN) to solve the binary offloading problem between the local and edge clouds in dynamic systems, and addresses the task assignment problem in a dynamic vehicular fog computing (VFC) environment. It simultaneously considers task priority, vehicle service availability, and computational resource sharing incentives, as well as using a soft actor–critic algorithm to maximize the utility of offloaded tasks.

In this paper, we consider adaptive user association, partial offloading, and resource allocation among multiple user devices and idle devices in a small area covered by a single BS. Unlike traditional algorithms considering latency and energy consumption, we maximize the computing power of the entire system. An improved algorithm based on DQN is presented to solve the proposed problem. The main contributions of this paper are summarized as follows:

- We construct a D2D-MEC framework that combines D2D communications and MEC technology. The user equipment with limited computational capability can offload part of its computation-intensive tasks to the MEC server located in the BS and the idle equipment nearby, and the allocation of computing resources is the responsibility of the BS. In order to maximize the computing power of the whole system under the condition of limited computing resources, we propose an MEC framework including partial offloading, resource allocation, and user association under the maximum delay constraint of the application.
- We propose an optimization problem with constraints on both delay and computational resources, which is NP hard. By analyzing the internal structure of the optimization problem, it is decomposed into two sub-problems. We prove that the optimal solutions of the two sub-problems constitute the optimal solution of the original problem. The convex optimization is employed to solve the optimal solution of the first subproblem. The second subproblem is described as a Markov decision process (MDP) used to maximize the amount of tasks calculated by the system, in which offload decisions, resource allocation, and user association are determined simultaneously. A DQN-based modeless reinforcement learning algorithm is proposed to maximize the objective function.
- Extensive simulations demonstrate that the proposed algorithm outperforms traditional MEC schemes, Q-learning, DQN, and other conventional algorithms under different system parameters.

The rest of this paper is organized as follows. In Section 2, we present the system model, including a network model, a channel model, a computation model, and problem formulation. In Section 3, we decompose the original problem into two subproblems. In Section 4, we propose a reinforcement learning algorithm based on DQN to solve subproblem 2. In Section 5, we show the simulation results. Finally, we conclude this study in Section 6.

## 2. System Model

### 2.1. Network Model

**Figure 1:**
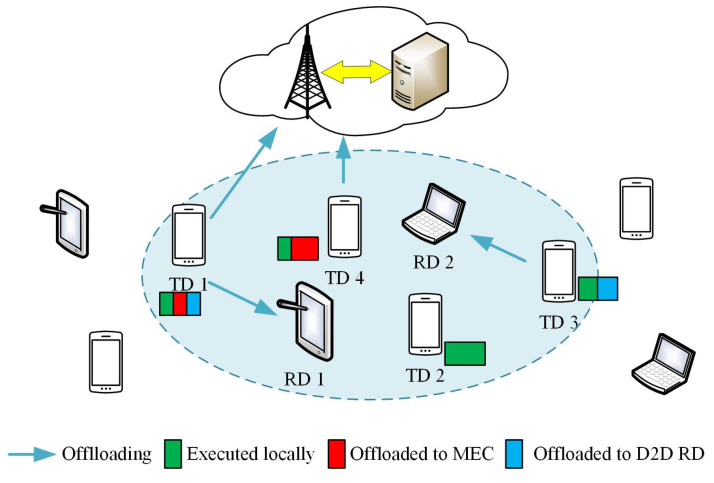
Network model.

As shown in Figure 1, the scenario we consider consists of a single BS equipped with an edge cloud server and mobile devices within the coverage area of the BS. A mobile device sends data to the BS through the cellular network and sends data to a nearby mobile device through D2D. Within the range of D2D communication, the mobile devices are divided into Task Devices (TDs), denoted as $U=\{u_{i}|i=1,2,\ldots ,U\}$, and D2D Resource Devices (D2D RDs), represented by $k_{i},i=1,2,\ldots ,K$. Set $K=\{k_{i}|i=0,1,2,\ldots ,K\}$ represents all resource devices, where $k_{0}$ is the BS. RDs can provide computing resources for tasks on TDs. The same as [31,32,33], the applications considered in this study are all oriented to data partitioning. The computing task on TD can be arbitrarily divided into three parts, and the computation is performed in parallel on the local, edge cloud, and a D2D RD at the same time.

We divide the system time into several time slots. The system state is constant in time slots, but changes between slots. Each slot BS allocates computing resources. The computing task on i∈U is denoted as $\phi_{i}=\left\{Q_{i},C_{i},\tau_{i},f_{i}\right\}$, where $Q_{i}$ is the size of the task data, $C_{i}$ indicates the number of CPU cycles per bit of data, representing the computational complexity of the application, $\tau_{i}$ is the maximum delay, and $f_{i}$ is the local computing capacity. Computing resources of $k_{i}\in K$ are denoted by $F_{i},i=0,1,2,\ldots ,K$.

### 2.2. Channel Model

We assume that the wireless channel state remains constant when each computation task is transmitted between the TD and RD. The transmission rate between the TD *i* and the BS is calculated by

(1) $$R_{ik_{0}}=B_{ik_{0}}log_{2}\left(1+\frac{p_{i}^{c}{\left|h_{ik_{0}}\right|}^{2}}{N_{0}}\right),\forall i\in U.$$

The transmission rate between TD *i* and D2D RD *j* is calculated by

(2) $$R_{ij}=B_{ij}log_{2}\left(1+\frac{p_{i}^{d}{\left|h_{ij}\right|}^{2}}{N_{0}}\right),\forall i\in U,\forall j\in K/k_{0}$$

where $h_{ij}$ is the channel power gain between TD *i* and RD *j*; $B_{ij}$ is the bandwidth allocated to the cellular channel or D2D channel between TD *i* and RD *j*; $p_{i}^{c}$ is the transmission power of cellular from TD *i* to BS; and $p_{i}^{d}$ is the transmission power of D2D from TD *i* to a D2D RD. Since the two powers are limited by the maximum uplink power $p_{i}^{max}$ of TD, they are subject to constraints:

(3) $$\begin{array}{cc} \; & 0\leq p_{i}^{c}\leq p_{i}^{max}, \end{array}$$

(4) $$\begin{array}{cc} \; & p_{i}^{d}=p_{i}^{max}-p_{i}^{c},\forall i\in U. \end{array}$$

### 2.3. Computation Model

The computing task on TD is divided into three parts, which are computed on a local, edge cloud, and D2D RD, respectively. $x_{ij}\in \left\{0,1\right\},\forall i\in U,\forall j\in K/k_{0}$ is the user association between TD *i* and RD *j*, $x_{ij}=1$ indicates that TD *i* offloads part of the computing task to D2D RD *j*, and otherwise, $x_{ij}=0$. Since a TD selects, at most, one D2D RD for computational offloading, there are constraints: $\sum_{j=k_{1}}^{k_{K}}x_{ij}\leq 1,\forall i\in U$. Let $\alpha_{i}\in \left[0,1\right],\beta_{i}\in \left[0,1\right],i\in U$ denote the proportion of a computing task on TD *i* that is offloaded to the edge cloud and D2D RD, respectively. Since the locally computed ratio should be non-negative, $\alpha_{i}$ and $\beta_{i}$ should satisfy the constraint: $0\leq \alpha_{i}+\beta_{i}\leq 1$. Let $f_{ij},\forall i\in U,\forall j\in K$ denote the computational resource allocated by RD *j* to TD *i*. Since RDs have limited computing resources, there are constraints: $\sum_{i=u_{1}}^{u_{U}}x_{ij}f_{ij}\leq F_{j},\forall j\in K$.

- **Local Computing:** The local computation delay of the task on TD *i* can be computed as
  (5) $$D_{i}^{l,c}=\frac{\left(1-\alpha_{i}-\beta_{i}\right)Q_{i}C_{i}}{f_{i}}.$$
- **Edge Computing:** The total latency of edge computing on TD *i* consists of three parts: (1) time for uploading computing tasks $D_{i}^{e,t}$, (2) time for executing tasks on the MEC server $D_{i}^{e,c}$, and (3) time for downloading computing results. Similar to [31,34], this study ignores the delay of sending results back to TDs from MEC server. This is because the size of the results is usually much smaller than the size of the transmitting data. Therefore, according to Equation (1), the delay of TD *i* to complete edge cloud computing can be computed as
  (6) $$D_{i}^{e}=D_{i}^{e,t}+D_{i}^{e,c}=\frac{\alpha_{i}Q_{i}}{R_{ik_{0}}}+\frac{\alpha_{i}Q_{i}C_{i}}{f_{ik_{0}}}.$$
- **D2D RDs Computing:** Similar to edge cloud computing, the delay of TD *i* to complete D2D RD computing can be obtained by (1) D2D transmission delay $D_{i}^{D,t}$ and (2) remote-execution delay $D_{i}^{D,c}$
  (7) $$D_{i}^{D}=D_{i}^{D,t}+D_{i}^{D,c}=\sum_{j=k_{1}}^{k_{K}}x_{ij}\left(\frac{\beta_{i}Q_{i}}{R_{ij}}+\frac{\beta_{i}Q_{i}C_{i}}{f_{ij}}\right).$$

Therefore, according to Equations (5)–(7), the total delay for completing the task $\phi_{i}$ on TD *i* is:

(8) $$D_{i}=\max\left\{D_{i}^{l,c},D_{i}^{e},D_{i}^{D}\right\}.$$

### 2.4. Problem Formulation

In this paper, we improve the computing capability of the whole system, and the computing capability of the system is reflected by the number of devices supported by the system. Under the constraints of computing resources and maximum delay, the number of devices served by the system indicates the computing capability of the system [25].

We regard the number of TDs that can complete the computing task as the optimization target, and $o_{u_{i}}=\left\{\begin{array}{cc} 1 & \mathrm{if}\;D_{i}\leq \tau_{i},\\ 0 & \mathrm{if}\;D_{i}>\tau_{i}, \end{array}\right.$ is the completion of the computing task on TD *i*. $o_{u_{i}}=1$ indicates that the task is completed within the maximum delay; otherwise, $o_{u_{i}}=0$. The list of notations is given in abbreviations part. The optimization problem of this study can be formulated as:

(9)P1:max{x,α,β,f}∑i=1Uoui,(10)s.t.xij∈{0,1},∀i∈U,∀j∈K/k0,(11)∑j=k1kKxij≤1,∀i∈U,(12)0≤αi≤1,∀i∈U,(13)0≤βi≤1,∀i∈U,(14)0≤αi+βi≤1,i∈U,(15)∑i=u1uUxijfij≤Fj,∀j∈K/k0(16)∑i=u1uUfik0≤F0,

where $x=\{x_{u_{1}k_{1}},x_{u_{1}k_{2}},\ldots ,x_{u_{1}k_{K}},\ldots ,x_{u_{U}k_{1}},x_{u_{U}k_{2}},\ldots ,x_{u_{U}k_{K}}\}$ denotes the user association vector between the TD and the D2D RD; $\alpha =\{\alpha_{u_{1}},\alpha_{u_{2}},\ldots ,\alpha_{u_{U}}\}$ is the offloading decision vector of edge cloud computing; $\beta =\{\beta_{u_{1}},\beta_{u_{2}},\ldots ,\beta_{u_{U}}\}$ is the offloading decision vector of D2D RDs computing; and $f=\{f_{u_{1}k_{0}},f_{u_{1}k_{1}},\ldots ,f_{u_{1}k_{K}},\ldots ,f_{u_{U}k_{0}},f_{u_{U}k_{1}},\ldots ,f_{u_{U}k_{K}}\}$ is the allocation decision of computing resources on RDs. Additionally, (10) indicates that the user-associated variable is a binary variable, $x_{ij}=1$ indicates that TD *i* offloads the task part to D2D RD *j*, or $x_{ij}=1$. (11) ensures that a TD can only select one of multiple D2D RDs devices for task offloading. Meanwhile, (12)–(14) indicate that the cloud, D2D RD, and local data ratios are all positive and cannot exceed one. Additionally, (15) and (16) ensure that RD *j* cannot allocate more computing resources to all TDs than its maximum computing capability.

**Theorem** **1.** *P1 is an NP-hard problem.*

**Proof.**  See Appendix A.    □

Theorem 1 shows that P1 is a NP-hard problem, and the object function of (9) is non-convex, which is a difficult problem to solve. In order to solve P1, we first decompose and simplify the problem, and then solve it using a reinforcement learning method instead of a conventional optimization method.

## 3. Problem Decomposition

We maximize the number of TDs served by the system under the constraints of limited computing resources. The requirement for the completion of tasks on TDs is that the computing time is less than the maximum delay. According to $D_{i}^{e,c}$ in Equation (6), the computing resource $f_{ik_{0}}$ allocated by the edge server to TD *i* is proportional to the data size $\alpha_{i}$. Similarly, according to $D_{i}^{D,c}$ in Equation (7), the computational resource $f_{ij}$ allocated by D2D RD *j* to TD *i* is proportional to the data size $\beta_{i}$. Based on the above analysis, reducing the value of $\alpha_{i}+\beta_{i}$ can alleviate computing resource TD *i*, which is occupied for the entire system. As a result, the system can have more computing resources to provide services for other TDs.

Based on the above analysis, in order to solve P1, we first determine the value of $\alpha_{i}+\beta_{i},\forall i\in U$ and the resource allocation scheme f, and then determine $\alpha_{i},\beta_{i}$ and user association x. Finally, we prove that the optimal solution obtained in this way is the same as the optimal solution of P1. Define variables $\gamma_{i}=\alpha_{i}+\beta_{i}$, $\alpha_{i}^{{}^{\prime }}=\frac{\alpha_{i}}{\alpha_{i}+\beta_{i}}$, $\beta_{i}^{{}^{\prime }}=\frac{\beta_{i}}{\alpha_{i}+\beta_{i}},\forall i\in U$. We have $\alpha_{i}=\gamma_{i}\alpha_{i}^{{}^{\prime }}$, $\beta_{i}=\gamma_{i}\beta_{i}^{{}^{\prime }}$, and $\beta_{i}^{{}^{\prime }}=1-\alpha_{i}^{{}^{\prime }}$. The variables in P1 change to $\left\{\gamma ,\alpha^{{}^{\prime }},x,f\right\}$.

In P2, the values of $\left\{\alpha^{{}^{\prime }},x\right\}$ are fixed, and the optimal {γ,f} are calculated. The goal of optimization is to minimize the demand for computing resources of the system. The mathematical formulation of the problem is expressed as

(17)P2:min{γ,f}∑j=k0kK∑i=u1uUxijfij,(18)s.t.0≤γi≤1,∀i∈U,(19)(1−γi)Cifi≤τi,∀i∈U,(20)γiαi′Qi(1Rik0+Cifik0)≤τi,∀i∈U,(21)γi(1−αi′)Qi∑j=k1kKxij(1Rij+Cifij)≤τi,∀i∈U,

where constraint (18) is set according to (14), and (19)–(21) represent constraints on local computing delay $D_{i}^{l,c}$, edge cloud computing delay $D_{i}^{e}$, and D2D computing delay $D_{i}^{D}$, respectively.

**Theorem** **2.** The optimal solution of P2 is given by $\left\{\gamma^{*},f^{*}\right\}$:

(22) $$\begin{array}{cc} \; & \gamma_{i}^{*}=1-\frac{\tau_{i}f_{i}}{C_{i}},\forall i\in U, \end{array}$$

(23) $$\begin{array}{cc} \; & {f_{ik_{0}}}^{*}=\frac{C_{i}\alpha_{i}^{{}^{\prime }}\gamma_{i}^{*}Q_{i}R_{ik_{0}}}{\tau_{i}R_{ik_{0}}-\alpha_{i}^{{}^{\prime }}\gamma_{i}^{*}Q_{i}},\forall i\in U, \end{array}$$

(24) $$\begin{array}{cc} \; & f_{ij}^{*}=\frac{C_{i}\gamma_{i}^{*}Q_{i}R_{ij}\left(1-\alpha_{i}^{{}^{\prime }}\right)}{\tau_{i}R_{ij}-\gamma_{i}^{*}\left(1-\alpha_{i}^{{}^{\prime }}Q_{i}\right)},\forall i\in U,\forall j\in K. \end{array}$$

**Proof.**  See Appendix B.    □

It can be observed that $\gamma_{i}^{*}$ is a constant independent of {x,α}, and $f_{ij}^{*}$ can be represented by variable $\alpha_{i}^{{}^{\prime }}$. Therefore, substituting the solution of P2 into P1 can obtain:

(25)P3:max{x,α′}∑i=1Uou1′,(26)s.t.xij∈{0,1},∀i∈U,j∈K/k0,(27)∑j=k1kKxij≤1,∀i∈U,(28)0≤αi′≤1,∀i∈U,(29)∑i=u1uUxijfij≤Fj,∀j∈K,

where $o_{u_{i}}^{{}^{\prime }}$ is obtained by

(30) $$\begin{array}{cc} \; & o_{u_{i}}^{{}^{\prime }}=\left\{\begin{array}{cc} 1 & \mathrm{if}\;\max\left\{D_{i}^{e*},D_{i}^{D*}\right\}\leq \tau_{i},\\ 0 & \mathrm{if}\;\max\left\{D_{i}^{e*},D_{i}^{D*}\right\}>\tau_{i}, \end{array}\right. \end{array}$$

(31) $$\begin{array}{cc} \; & D_{i}^{l,c*}=\frac{\left(1-\gamma_{i}^{*}\right)C_{i}}{f_{i}}=\tau_{i} \end{array}$$

(32) $$\begin{array}{cc} \; & D_{i}^{e*}=\gamma_{i}^{*}\alpha_{i}^{{}^{\prime }}Q_{i}\left(\frac{1}{R_{ik_{0}}}+\frac{C_{i}}{{f_{ik_{0}}}^{*}}\right), \end{array}$$

(33) $$\begin{array}{cc} \; & D_{i}^{D*}=\gamma_{i}^{*}\left(1-\alpha_{i}^{{}^{\prime }}\right)Q_{i}\sum_{j=k_{1}}^{k_{K}}x_{ij}\left(\frac{1}{R_{ij}}+\frac{C_{i}}{f_{ij}^{*}}\right). \end{array}$$

**Theorem** **3.** The optimal solutions $\left\{\gamma^{*},f^{*}\right\}$,$\left\{x^{*},\alpha^{{}^{\prime }*}\right\}$ obtained by P2 and P3 are the optimal solutions of P1.

**Proof.**  See Appendix C.    □

## 4. DQN-Based Computation Offloading

In order to solve problem P3, we express it as a MDP, which can be solved using model-free reinforcement learning, and propose an improved reinforcement learning algorithm based on DQN. Compared with the conventional DQN, the improved reinforcement learning algorithm can enable the agent to learn a better solution and converge to solve the problems. In this paper, we improve the number of task users that the system can serve by optimizing the offloading strategy and the allocation of computing resources in the system. Obviously, the optimal solution is unknown, so the agent can easily fall into a sub-optimal solution after finding a feasible allocation scheme and obtaining a positive reward. Therefore, in order to make the agent constantly search for a better solution, we compare and update the recorded action trajectory during the learning process, and then make random selection with a certain probability under the condition that the agent learns a lot of the recorded action trajectory, so that the agent has the opportunity to learn a better solution. In addition, we set the reward value given by the environment when learning the recorded action trajectory. This is different from using the Q-network to decide the action, which is used for fast convergence.

We call the proposed algorithm DQN-PTR, which integrates the priority action trajectory replay method into DQN. In this section, we first define the three key elements of the MDP problem, i.e., the state space, action space, and reward function. Then, we explain the detailed implementation of our proposed algorithm for reinforcement learning.

### 4.1. Three Key Elements for MDP

- State Space:
  At each time slot, the agent observes and collects all device information within the range of the BS. At step *t*, the state of the system consists of two parts: $s_{t}=\left\{F\left(t\right),\phi \left(t\right)\right\}$. Among them, $F=\{F_{0}\left(t\right),F_{1}\left(t\right),\ldots ,F_{K}\left(t\right)\}$ represents the computing resources of all resource devices in the system, and $\phi \left(t\right)=\left\{\phi_{u_{1}},\phi_{u_{2}},\ldots ,\phi_{u_{U}}\right\}$ where $\phi_{i}\in \left\{0,1\right\},i\in U$ represents the completion of the tasks on the task devices. We define $s_{0}$ as the system state observed by the BS at the beginning of the time slot; that is, $s_{0}=\left\{F_{0},F_{1},\ldots ,F_{K},0,0,\ldots ,0\right\}$.
- Action Space:
  The action space consists of two parts: $A_{t}=\left\{x,\alpha^{{}^{\prime }}\right\}$, where $x=\{x_{u_{1}},x_{u_{2}},\ldots ,x_{u_{U}}\}$, $x_{i}=\left\{x_{ik_{1}},x_{ik_{2}},\ldots ,x_{ik_{K}}\right\},i\in U$ represents the offload association between the TDs and the D2D RDs. $\alpha^{{}^{\prime }}$ is the task offload ratio of the currently assigned TDs. According to constraint (26) and (27), it is stipulated that action $a_{t}$ at step *t* satisfies condition ∑x(t)=1.
- Reward Function:
  The objective function of P3 is the sum of the TD devices that complete the calculation. Considering that the size of the computing tasks of each device is different, to ensure the fairness of the evaluation, the reward function is defined as the sum of the size of the computing tasks completed in the current time slot:
  (34) $$R\left(s_{t},a_{t}\right)=\left\{\begin{array}{cc} \sum_{i=1}^{U}o_{u_{i}}Q_{i}, & \mathrm{if}\;\sum_{i=1}^{U}o_{u_{i}}>0,\\ -1, & else, \end{array}\right.$$

In the reinforcement learning process, in each episode, as the environment performs the *t* step action, the state and action space of the next step also change. Assume that the action selected by the agent at step *t* is $a_{t}=\left\{x_{ij}=1,\alpha^{{}^{\prime }}\left(t\right)\right\}$. $a_{t}$ is executed by the environment when $s_{t},a_{t}$ satisfy the condition, given by

(35) $$\begin{array}{cc} \; & \phi_{i}=0, \end{array}$$

(36) $$\begin{array}{cc} \; & Q_{i}C_{i}\gamma_{i}\alpha^{{}^{\prime }}\left(t\right)\leq F_{j}\left(t\right), \end{array}$$

(37) $$\begin{array}{cc} \; & Q_{i}C_{i}\gamma_{i}\left(1-\alpha^{{}^{\prime }}\left(t\right)\right)\leq F_{0}\left(t\right). \end{array}$$

With the successful execution of $a_{t}$, the remaining resources of the RDs and the TDs that have not allocated computing resources are reduced, and are given by

(38) $$\begin{array}{cc} \; & F_{j}\left(t+1\right)=F_{j}\left(t\right)-Q_{i}C_{i}\gamma_{i}\alpha^{{}^{\prime }}\left(t\right), \end{array}$$

(39) $$\begin{array}{cc} \; & F_{0}\left(t+1\right)=F_{0}\left(t\right)-Q_{i}C_{i}\left(1-\gamma_{i}\alpha^{{}^{\prime }}\left(t\right)\right), \end{array}$$

(40) $$\begin{array}{cc} \; & A_{t+1}=A_{t}/x_{i}. \end{array}$$

If any of the conditions (35)–(37) are not met, $a_{t}$ is infeasible, i.e., $s_{t+1}=s_{t}$, $A_{t+1}=A_{t}$.

### 4.2. Algorithm Design Based on DQN

**Figure 2:**
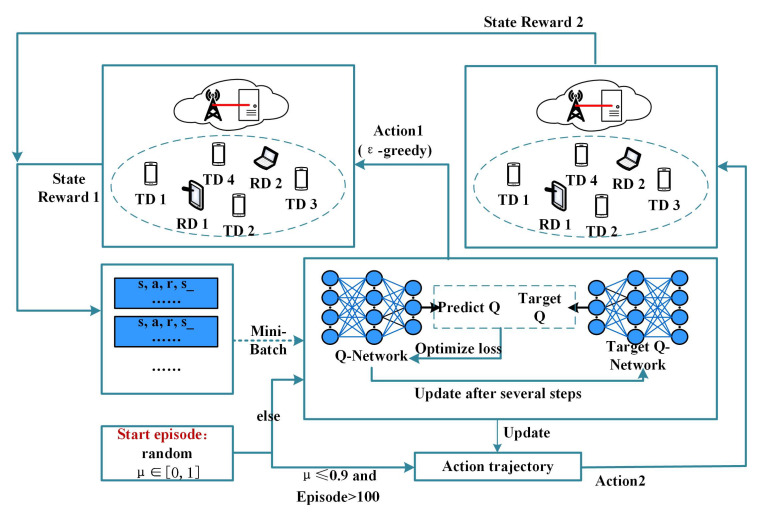
DQN-PTR based MEC system.

The structure of the DQN-PTR algorithm proposed in this paper is shown in Figure 2, which mainly includes four parts: the environment, the replay buffer, the networks, and the trajectory record. The environment performs actions, computes rewards, and gives transitions to the state and the action space. The replay buffer stores the task offloading experiences, which are used to train the Q-Network. The network part includes two networks, which are used to predict the Q value and target the Q value, respectively. Network parameters are updated according to the differences between the two Q values. The recorded action trajectory is updated at the end of each training episode. If the result of the current episode is better than the recorded result, the action trajectory of the current episode is used to replace the originally recorded action trajectory.

We propose an DQN-PTR-based task offloading algorithm in Algorithm 1, and explain the main steps in detail, as follows:

1. Initialize the action-value function Q(s,a) and the target action-value function $\hat{Q}\left(s,a\right)$ with parameters θ and $\theta^{{}^{\prime }}$, respectively. Initialize experiment replay buffer *D* to an empty set of size *N*.
2. Initialize ϵ=0, and specify that the growth rate of ϵ is $\epsilon_{increment}=0.0001$ and grows to $\epsilon_{max}=0.9999$. This parameter determines the probability of random selection when the agent selects an action, and the probability of random selection decreases with the update of the network parameters.
3. Initialize the optimal trajectory record O=⌀ and the maximum total return value R=0.
4. In each episode, the agent in the BS collects the $s_{0}$. Initialize $R^{{}^{\prime }}=0$ to calculate the total return value of this episode. μ∈[0,1] is used to determine whether all actions in this episode are determined by the Q-Network or the optimal trajectory.
5. If the training episode is less than or equal to 100, ϵ is used to decide whether the choice of action is randomly selected or selected according to the maximum *Q* value.
6. When the training episode is greater than 100: μ>0.9, the selection of actions in this episode is the same as that in point number five; when μ≤0.9, the actions are performed in accordance with the optimal trajectory O in this episode. It should be noted that the reward settings in environment 1 and environment 2 are different. They are expressed as follows.
  $$\begin{array}{cc} Environment\;1:\; & R\left(s_{t},a_{t}\right)=\left\{\begin{array}{cc} \sum_{i=1}^{U}o_{u_{i}}Q_{i}, & \mathrm{if}\;\sum_{i=1}^{U}o_{u_{i}}>0,\\ -1, & else, \end{array}\right.\\ Environment\;2:\; & R\left(s_{t},a_{t}\right)=\left\{\begin{array}{cc} \sum_{i=1}^{U}o_{u_{i}}Q_{i}, & \mathrm{if}\;\sum_{i=1}^{U}o_{u_{i}}>0.\\ 0, & else. \end{array}\right. \end{array}$$
7. When step *t* ends, store $\left(s_{t},a_{t},r_{t},s_{t+1}\right)$ in the experience replay buffer; update the total reward value of the current cycle $R^{{}^{\prime }}=R^{{}^{\prime }}+r_{t}$; retrieve multiple records from the experience replay buffer to update Q-Network; increase the value of ϵ.
8. At the end of each episode, compare the values of *R* and $R^{{}^{\prime }}$, and compare the number of training steps in this period with the length of O to determine whether to update the action trajectory record.

The advantages of the improved DQN-PTR includes:

- When adding a judgment item to the outer layer of the traditional DQN, the setting of the reward function becomes flexible.
- In addition to the traditional DQN replay memory *D*, a new cache space O is added, which can be used to record the excellent action trajectory.
- Depending on the type of problem solved, the action space of the DQN-PTR can vary with the execution of each action.

**Algorithm 1:** DQN-PTR to solve P301: Initialize the Q-Network *Q* with random weights θ02: Initialize the Target Q-Network $\hat{Q}$ with weights $\theta^{{}^{\prime }}=\theta$03: Initialize replay memory *D* to capacity *N*04: Initialize $\epsilon =0,\epsilon_{increment}=0.0001,\epsilon_{max}=0.9999$05: Initialize optimal trajectory O to empty and maximum total return R=006: **For** episode=1,M **do**07:  Initialize sum reward $R^{{}^{\prime }}=0$08:  Initialize state $s_{0}$, μ=rand∈[0,1]09:  **For** each step *t* **do**10:   **If** episode≤100 **or** μ>0.9 **then**11:    **If** rand∈[0,1]≥ϵ **then**12:     Select a random action $a_{t}$13:    **else**14:     Set $a_{t}=arg\max_{a}Q\left(s_{t},a;\theta \right)$15:    **end if**16:    Execute action $a_{t}$, observe next state $s_{t+1}$ and reward $r_{t}$ according to environment 117:   **else**18:    Set $a_{t}$ according O[t]19:    Execute action $a_{t}$, observe next state $s_{t+1}$ and reward $r_{t}$ according to environment 220:   **end if**21:   $R^{{}^{\prime }}=R^{{}^{\prime }}+r_{t}$22:   Store transition $\left(s_{t},a_{t},r_{t},s_{t+1}\right)$ in *D*23:   **If** episode terminates at step t+1 **then**24:    **If** $R^{{}^{\prime }}>R$ **then**25:     Replace the trajectory in O with $\left\{a_{1},\ldots ,a_{t}\right\}$26:     $R=R^{{}^{\prime }}$27:    **else if** $R^{{}^{\prime }}=R$ and t<len(O) **then**28:     Replace the trajectory in O with $\left\{a_{1},\ldots ,a_{t}\right\}$29:    **end if**30:    **break**31:   **end if**32:   Sample random mini-batch of transitions $\left(s_{i},a_{j},r_{j},s_{j+1}\right)$ from *D*33:   Set $y_{j}=\left\{\begin{array}{cc} & r_{j},\;\mathrm{if}\;\mathrm{episode}\;\mathrm{terminates}\;\mathrm{at}\;\mathrm{step}j+i,\\ & r_{j}+\gamma \max_{a^{{}^{\prime }}}\hat{Q}\left(s_{j+1},a^{{}^{\prime }};\theta^{{}^{\prime }}\right),\;\mathrm{otherwise}, \end{array}\right.$34:   Perform a gradient descent step on ${\left(y_{j}-Q\left(s_{j},a_{j};\theta \right)\right)}^{2}$ with respect to the network parameters θ35:   **If** $\epsilon <\epsilon_{max}$ **then**36:    $\epsilon =\epsilon +\epsilon_{increment}$37:   **end if**38:   Every *C* steps reset $\hat{Q}=Q$39:  **end for**40: **end for**

## 5. Analysis of Simulation Results

In this section, we evaluate the performance of the computational offloading scheme proposed in this paper and the DQN-PTR algorithm through computer simulations. We first present the simulation parameters of the system. Then, we discuss the experimental results.

### 5.1. Simulation Setup

In the simulation, we assume the following scenario. We consider partial offloading between multiple devices in a small area covered by a single base station.The edge server on the base station side is equipped with an reinforcement learning agent which can make decisions about the offloading scheme in this area. In addition, the computing resources in the system are limited, and computing tasks cannot be completed locally. According to references [7,25,26,29,34,35], we set the simulation parameters to match our research scenario. We consider that the transmission power, channel bandwidth, and background noise of each device are 2 W, 10 MHz, and −170 dBm. The computing capacity of TDs and RDs are 24 Mcycles/s and 35 Mcycles/s, respectively. The data size and maximum latency of each task are 2.15 Mbits and 1 s, respectively. The number of CPU cycles is set 20 Cycles/bit.

We compare the proposed algorithm with traditional MEC schemes, two benchmark algorithms, and reinforcement learning algorithms:

- *Full Local*: All TDs execute their tasks via local computing.
- *Local-cloud*: The computing tasks on TDs can be divided into two parts, which are computed on the local and edge cloud, respectively. In order to make full use of computing resources, all computing resources on TD *i* are allocated to task $\phi_{i}$. If the local resources are insufficient, the computing resources will be supplemented by the edge cloud.
- *RBA*: The TD *i* randomly selects a D2D RD device for computing offload and utilizes all the computing resources of the D2D RD device. If the computing resources of the two places are insufficient, the edge cloud will supplement the computing resources.
- *GBA*: The TD *i* selects the D2D RD device with the largest remaining resources. Under the condition of making full use of local computing resources, the remaining computing tasks are evenly sent to the D2D RD device and edge cloud for computing.
- *Q-learning*: Q-learning is a basic reinforcement learning algorithm. Using Q-learning to solve P3, in simulation, the state space, action space, and reward function of Q-learning are all the same as those in DQN-PTR.
- *DQN*: DQN is an improved reinforcement learning algorithm based on Q-learning. In the simulation, the state space, action space, and reward function of DQN are all the same as those in DQN-PTR.

### 5.2. Simulation Result

**Figure 3:**
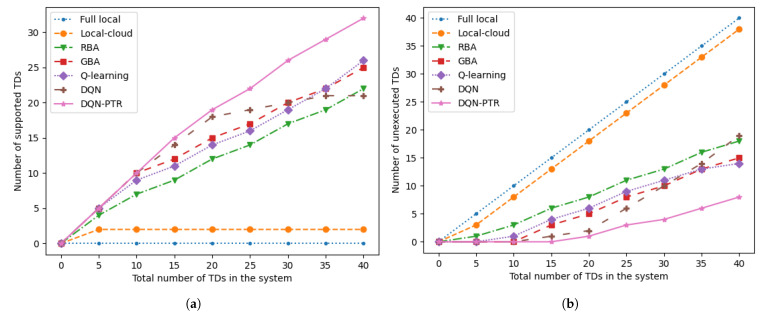
(**a**) The number of supported TDs versus the total number of TDs in the system. (**b**) The number of unexecuted TDs versus the total number of TDs in the system.

In Figure 3, we show the number of supported (or unexecuted) TDs versus the total number of TDs in the system. The computing capacity of the MEC server is 50 Mcycles/s, and the number of D2D RDs is $\frac{1}{2}$ of the number of TDs. Since the scenarios we study mainly concern computationally intensive tasks, none of the tasks are computed locally. Relying on the computing resources of the local and MEC server, when the number of TDs reaches five, the upper limit of the system computing capacity can be reached. When considering D2D RDs in the system, the total number of computing tasks that the system can complete naturally increases as the number of TDs increases. In Figure 3, the DQN-PTR method proposed in this paper can achieve the best results. The DQN algorithm works satisfactorily when the number of devices is relatively small, but the results become worse as the number increases. However, the gap between Q-learning and DQN-PTR is always large. This is because as the number of devices increases, the number of actions and states in reinforcement learning also increases, which results in worse training results under the same training episodes. Additionally, when the number of TDs is greater than 10, the results obtained by the RBA and GBA algorithms are both smaller than the algorithm proposed in this paper.

**Figure 4:**
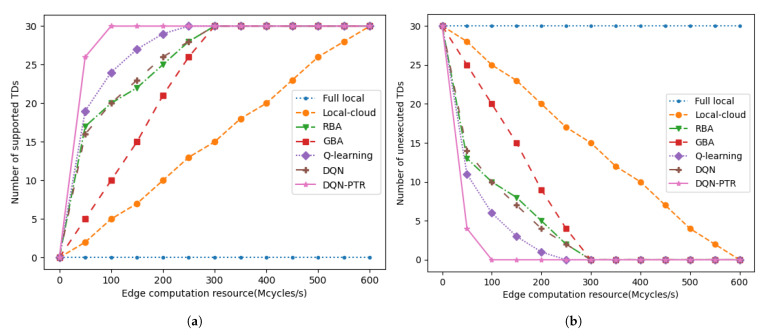
(**a**) The number of supported TDs versus the edge computation resource. (**b**) The number of unexecuted TDs versus the edge computation resource.

In Figure 4, we show the number of supported (or unexecuted) TDs as the computing resources of the MEC server increases. The number of TDs and D2D RDs in the system is 30 and 15, respectively. It can be observed from Figure 4 that if the DQN-PTR algorithm is used for computing offload planning, the MEC server only needs to provide 100 Mcycles/s of computing resources to compute all tasks in the system. In addition, if the computing resources of D2D RDs in the system are utilized, even the relatively poor allocation algorithm (DQN, RBA and GBA) can save about 300 Mcycles/s of cloud computing resources compared with traditional local-cloud offloading.

**Figure 5:**
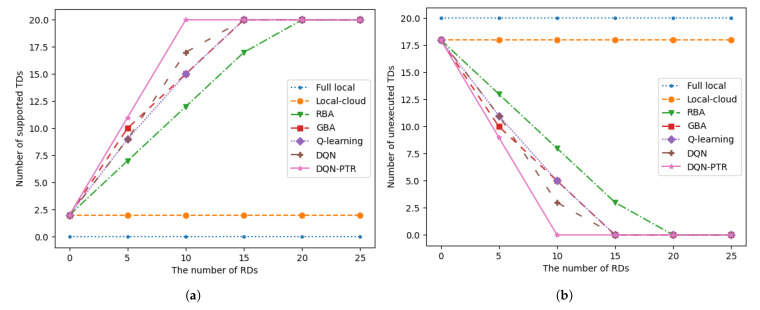
(**a**) The number of supported TDs versus the number of RDs. (**b**) The number of unexecuted TDs versus the number of RDs.

In Figure 5, we present the number of supported (or unexecuted) TDs versus the number of D2D RDs. The computing power of the MEC server is fixed at 50 Mcycles/s and the number of TDs is 20, so the total data volume of all tasks in the system is 43.00 Mbits. As can be seen from Figure 5, our proposed DQN-PTR algorithm requires the fewest D2D RD devices. DQN, GBA, and Q-learning algorithms require 15 D2D RD devices to complete all tasks, while RBA requires 20 D2D RD devices.

**Figure 6:**
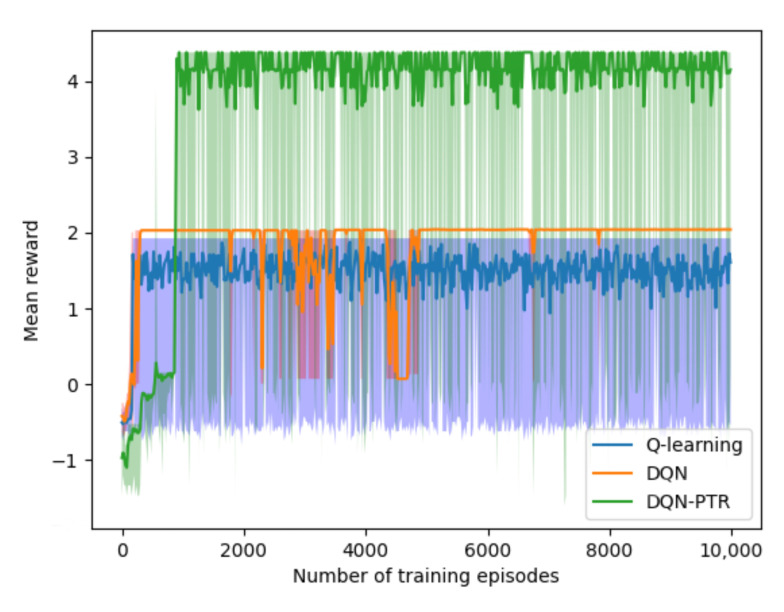
Learning curves of Q-learning (blue), CQN (orange) and DQN-PTR (green).

Figure 6 shows the learning curves of Q-learning, DQN, and DQN-PTR. The number of TDs and D2D RDs is 20 and 10, respectively. In order to ensure fairness, the three algorithms have the same parameters, i.e., the maximum number of steps allowed per episode is 50. The ε−greedy value increases from 0 to 0.95 with a growth rate of 0.0001. Learning rate γ, replay memory size, and mini-batch size are 0.0001, 10,000, and 200, respectively. Reference [36] for an analysis of our curves, we found that DQN-PTR performed better than DQN and Q-learning. Although Q-learning has the fastest learning speed, our proposed algorithm is more stable than DQN in terms of the fluctuation of the curve, and obtains the highest average return value.

## 6. Conclusions

This paper has proposed an integrated framework for multi-user partial offloading and resource allocation, combining MEC and D2D technologies. Under this framework, we can make the decision of computational offloading and the resource allocation of MEC and idle devices. We have also designed a convex optimization method to simplify the problem. Finally, we have derived a DQN-based reinforcement learning algorithm to solve the problem. Simulation results have shown that the proposed scheme has better performance than other benchmark schemes under different system parameters.

## Data Availability

Not applicable.

## Abbreviations

The following abbreviations are used in this manuscript:

MEC	Mobile edge computin
D2D	Device-to-device
MDP	Markov decision process
DQN	Deep Q network
AR	Augmented reality
VR	Virtual reality
UE	User equipment
BS	Base station
SCA	Successive convex approximation
GP	Geometric programming
DCN	Distributed computing node
MIP	Mixed integer programming
RL	Reinforcement learning
VFC	Vehicular fog computing
TD	Task device
RD	Resource device
D2D RD	D2D Resource device
Notations
$u_{i}$	The index of the TD *i*
$k_{i}$	The index of the RD *i*, where $k_{0}$ represents the BS, and the others represent D2D RD
U	The number of TDs
K	The number of D2D RDs
U	The set of all TDs
K	The set of all RDs
$\phi_{i}$	The index of the computing task on i∈U
$Q_{i}$	The data size of the task $\phi_{i}$
$C_{i}$	CPU cycles per bit required for task $\phi_{i}$
$\tau_{i}$	The maximum delay of the task $\phi_{i}$
$f_{i}$	The local computing capacity of the TD *i*
$F_{i}$	The computing resource of the RD *i*
$R_{ij}$	The transmission rate between the TD *i* and the RD *j*
$B_{ij}$	The bandwidth allocated to the channel between TD *i* and RD *j*
$p_{i}^{c}$	The cellular transmission power from TD *i* to BS
$p_{i}^{d}$	The transmission power of D2D from TD *i* to a D2D RD
$p_{i}^{max}$	The maximum uplink power of TD *i*
$x_{ij}$	User association between TD *i* and RD *j*
$\alpha_{i}$	The proportion of a computing task on TD *i* that is offloaded to the BS
$\beta_{i}$	The proportion of a computing task on TD *i* that is offloaded to D2D RD
$D_{i}^{l,c}$	The local computation delay of the task on TD *i*
$D_{i}^{e}$	The delay of TD *i* to complete edge cloud computing
$D_{i}^{e,t}$	Cellular transmission delay of TD *i*
$D_{i}^{e,c}$	The computation delay of task $\phi_{i}$ at BS
$D_{i}^{D}$	The delay of TD *i* to complete D2D RD computing
$D_{i}^{D,t}$	D2D transmission delay of TD *i*
$D_{i}^{D,c}$	The computation delay of task $\phi_{i}$ at D2D RD
$D_{i}$	The total delay for completing the task $\phi_{i}$ on TD *i*
$o_{u_{i}}$	The completion of the computing task on TD *i*

## Appendix A

Considering that when the MEC server and all TDs do not provide computing resources, and the transfer time is fast enough. In this case, the number of computing tasks is much more than the number of D2D RDs, and satisfies:

$$\begin{array}{cc} \; & \phi_{i}=\phi_{U},\forall i\in U,\\ \; & F_{i}=F_{K},\forall j\in K/k_{0}. \end{array}$$

Therefore, the maximum number of tasks supported by a single D2D RD is $\frac{F_{K}}{Q_{i}C_{i}\tau_{i}}$. In P1, the problem of maximizing the number of tasks can be rewritten as

$$\begin{array}{cc} PA:\; & \max_{x}\sum_{j=k_{1}}^{k_{K}}\left(\sum_{i=u_{1}}^{u_{U}}x_{ij}\right),\\ s.t.\; & x_{ij}\in \left\{0,1\right\},\forall i\in U,\forall j\in K/k_{0},\\ \; & \sum_{j=k_{1}}^{k_{K}}x_{ij}=1,\forall i\in U,\\ \; & \sum_{i=u_{1}}^{u_{U}}x_{ij}\leq \frac{F_{K}}{Q_{i}C_{i}\tau_{i}},\forall j\in K/k_{0}. \end{array}$$

It is difficult to obtain the optimal solution of the problem PA. For the fact that x is binary, then the feasible set is not convex, PA is non-convex integer programs. According to the statement in [17,37,38], PA is NP-hard. From [39], P1 is also NP-hard because P1 is a special case of PA.

## Appendix B

According to (10) and (11), $\sum_{j=k_{1}}^{k_{K}}x_{ij}$ is equal to 0 or 1. Therefore, in order to prove Theorem 1, the problem is classified according to the value of user association x.

- When $\sum_{j=k_{1}}^{k_{K}}x_{ij}=0$, the computing task of TD *i* is only computed at the local and edge cloud, thus $\beta_{i}^{{}^{\prime }}=0$,$f_{ij}=0,\forall j\in K/k_{0}$ and $\alpha_{i}^{{}^{\prime }}=1$. In this case, constraint (21) is obviously satisfied. We then have
  (A1) $$\gamma_{i}\geq 1-\frac{\tau_{i}f_{i}}{C_{i}}.$$
  From (20), we can obtain
  (A2) $$f_{ik_{0}}\geq \frac{C_{i}\alpha_{i}^{{}^{\prime }}\gamma_{i}Q_{i}R_{ik_{0}}}{\tau_{i}R_{ik_{0}}-\alpha_{i}^{{}^{\prime }}\gamma_{i}Q_{i}}.$$
  Take the derivative of the right hand side of the above inequality, we have
  (A3) $$\frac{d}{d\gamma_{i}}\left(\frac{C_{i}\alpha_{i}^{{}^{\prime }}\gamma_{i}Q_{i}R_{ik_{0}}}{\tau_{i}R_{ik_{0}}-\alpha_{i}^{{}^{\prime }}\gamma_{i}Q_{i}}\right)=\frac{C_{i}\alpha_{i}^{{}^{\prime }}Q_{i}{R_{ik_{0}}}^{2}\tau_{i}}{{\left(\tau_{i}R_{ik_{0}}-\alpha_{i}^{{}^{\prime }}\gamma_{i}Q_{i}\right)}^{2}}>0.$$
  To sum up, when the equal signs of (A1) and (A2) hold, $f_{ik_{0}}$ can take the minimum value, that is, (22) and (23) are proved.
- When $\sum_{j=k_{1}}^{k_{K}}x_{ij}=1$, it means that TD *i* offloads the computing task to a certain D2D RD, we define the D2D RD as D2D RD *j*. According to (21), we have
  (A4) $$\begin{array}{cc} \; & \gamma_{i}\left(1-\alpha_{i}^{{}^{\prime }}\right)Q_{i}\left(\frac{1}{R_{ij}}+\frac{C_{i}}{f_{ij}}\right)\leq \tau_{i}, \end{array}$$
  (A5) $$\begin{array}{cc} \; & f_{ij}\geq \frac{C_{i}\gamma_{i}Q_{i}R_{ij}\left(1-\alpha_{i}^{{}^{\prime }}\right)}{\tau_{i}R_{ij}-\gamma_{i}\left(1-\alpha_{i}^{{}^{\prime }}\right)Q_{i}}. \end{array}$$
  Similar to (A2), when the equal signs of (A1) and (A5) hold, $f_{ij}$ takes the minimum value, that is, (24) can be proved.
- When $\sum_{j=k_{1}}^{k_{K}}x_{ij}=0$, $\alpha_{i}^{{}^{\prime }}=1$ is substituted into (24) and the result is $f_{ij}^{*}=0$. Therefore, Theorem 1 is proved in both cases.

## Appendix C

In order to prove that the optimal solutions of P2 and P3 are the same as the optimal solutions of P1, it is necessary to prove that the maximum values of the objective functions calculated by the optimal solutions obtained by P3 and P1 are the same. To this end, we first denote the optimal solution set of P2 and P3 as: $\left\{\gamma^{*},f^{*},x^{*},\alpha^{{}^{\prime }*}\right\}$. The maximum value of the objective function of P3 is $N_{3}$. The optimal solution set of P1 is set as: $\left\{x_{1}^{*},\alpha_{1}^{*},\beta_{1}^{*},f_{1}^{*}\right\}$, where $\gamma_{1}^{*}=\alpha_{1}^{*}+\beta_{1}^{*}$,$\alpha_{1}^{{}^{\prime }*}=\frac{\alpha_{1}^{*}}{\gamma_{1}^{*}}$,$\beta_{1}^{*}=\gamma_{1}^{*}-\alpha_{1}^{{}^{\prime }*}$, and the maximum value of the objective function is $N_{1}$.

From (18), (26)–(28), it is easy to know that $\gamma^{*},x^{*},\alpha^{{}^{\prime }*}$ satisfy (10)–(14), and from (29), it can be deduced that $f^{*}$ calculated by $\alpha^{{}^{\prime }*}$ satisfies (). To sum up, $\left\{\gamma^{*},f^{*},x^{*},\alpha^{{}^{\prime }*}\right\}$ are feasible solutions of P1, from which $N_{3}\leq N_{1}$ can be deduced.

Similarly, it can be proved that $\left\{x_{1}^{*},\alpha_{1}^{*},\beta_{1}^{*},f_{1}^{*}\right\}$ satisfy the constraints (18), (26)–(29). Assuming that the conditions (19)–(21) are not satisfied, it means that there are offloading schemes $\left\{x_{1i}^{*},\alpha_{1i}^{*},\beta_{1i}^{*},f_{1i}^{*}\right\}$ of $TD_{i},i\in U$ that do not satisfy the constraints ()–(), that is, $D_{i}>\tau_{i},o_{u_{i}}=0,f_{1i}^{*}\leq 0,0\leq \alpha_{1i}^{*}+\beta_{1i}^{*}\leq 1$. This means that the TD *i* and all computing resources $f_{1i}^{*}=\left\{f_{1ij}^{*}\right\},\forall j\in K$ allocated to TD *i* does not affect the value of the objective function. Group all TDs that do not satisfy the constraints into set $M=\{m_{i}|i=1,2,\ldots ,M\}$. Set $N=U/M=\{n_{i}|i=1,2,\ldots ,U-M\}$ and redefine the computing resource in the system as $F_{j}^{{}^{\prime }}=F_{j}-\sum_{i=1}^{M}f_{1m_{i}j}^{*},j\in K$. Re-resource allocation is performed on TDs set N and network resource state $F_{j}^{{}^{\prime }}$ through P1, and the obtained resource allocation scheme is equivalent to $\left\{x_{1}^{*},\alpha_{1}^{*},\beta_{1}^{*},f_{1}^{*}\right\}$ and both satisfy the constraints of P2 and P3. So the maximum value of the objective function is still $N_{1}$ and the solution is a feasible solution of P3. The maximum objective function obtained by solving the same problem through P2,P3 is defined as $N_{3}^{{}^{\prime }}$, so $N_{1}\leq N_{3}^{{}^{\prime }}$. In addition, since the number of TDs and the computing resources in the network are both reduced, the obtained maximum objective function value $N_{3}^{{}^{\prime }}\leq N_{3}$, so $N_{1}\leq N_{3}^{{}^{\prime }}\leq N_{3}$.

To sum up, $N_{1}\leq N_{3}^{{}^{\prime }}\leq N_{3}$ and $N_{3}\leq N_{1}$, so there can only be $N_{3}=N_{1}$. Theorem 2 is proved.
